# Supplementary material for: ATP Synthase Subunit Epsilon Overexpression Promotes Metastasis by Modulating AMPK Signaling to Induce Epithelial-to-Mesenchymal Transition and Is a Poor Prognostic Marker in Colorectal Cancer Patients
Source: J Clin Med. 2019 Jul 21;8(7):1070. doi: 10.3390/jcm8071070 (PMC6678251; doi:10.3390/jcm8071070)
Supplement: Supplementary file 1 [file jcm-08-01070-s001.pdf]

Table S1. The demography of patients with Colorectal Cancer

	Case/Total	%
Age		
21-92 Years, Median 70 Years	243/243	100.00
Sex		
Male	138/243	56.79
Female	107/243	44.03
ATP5E (Score)		
-	49/243	20.16
+	58/243	23.87
++	79/243	32.51
+++	57/243	23.46
p-AMPKα thr 172 (Score)		
-	72/205	35.12
+	46/205	22.44
++	60/205	29.27
+++	27/205	13.17
E-cadherin (Score)		
-	18/210	8.57
+	35/210	16.67
++	86/210	40.95
+++	71/210	33.81
Location		
Ascending Colon	33/243	13.58
Ascending/Transverse Colon	1/243	0.41
Cecum	10/243	4.12
Descending Colon	17/243	7.00
Rectum	81/243	33.33
Rectum/Sigmoid Colon	11/243	4.53
Sigmoid Colon	61/243	25.10
Transverse Colon	26/243	10.70
Transverse/ Descending Colon	1/243	0.41
Not Available	2/243	0.82
Histological Type		
Adenocarcinoma	199/243	81.89
Adenocarcinoma with Focal Mucin	23/243	9.47
Adenocarcinoma with Focal Squamous	1/243	0.41
Mucinous Adenocarcinoma	20/243	8.23
pT (Tumor Status)		
pT1	9/243	3.70
pT2	43/243	17.70
pT3	164/243	67.49
pT4	27/243	11.11

Table S2. The relationship between ATP5E expression and the clinical-pathological characteristics of colorectal cancer

	ATP5E Expression		
Characteristics	Low (0,1) (n =107)	High (2,3) (n =136)	Chi-Square (Yates)
Age			0.09
≦59	32 (29.91 %)	24 (17.65 %)	
60-69	28 (26.17 %)	29 (21.32 %)	
≧70	47 (43.93 %)	83 (61.03 %)	
Sex			1.3
Male	55 (51.40 %)	81 (59.56 %)	
Female	52 (48.60 %)	55 (40.44 %)	
pT (Tumor Status)			1.15
pT 1-2	19 (17.76 %)	33 (24.26 %)	
pT 3-4	88 (82.24 %)	103 (75.74 %)	
pN (Lymph Node Status)			0.08
pN 0	54 (50.47 %)	65 (47.79 %)	
pN 1-2	53 (49.53 %)	71 (52.21 %)	
pM (Distal Metastasis)			0.69
pM0	96 (89.72 %)	116 (85.29 %)	
pM1	11 (10.28 %)	20 (14.71 %)	
Stage (AJCC)			0.46
I-II	52 (48.60 %)	59 (43.38 %)	
III-IV	55 (51.40 %)	77 (56.62 %)	
Recurrence			2.67
No	91 (85.05 %)	103 (75.74 %)	
Yes	16 (14.95 %)	33 (24.26 %)	

*, All P values were generated under df=1. p value<0.05 was considered statistically significant (Student’s t-test for continuous variables and Pearson’s chi-square test for variables).

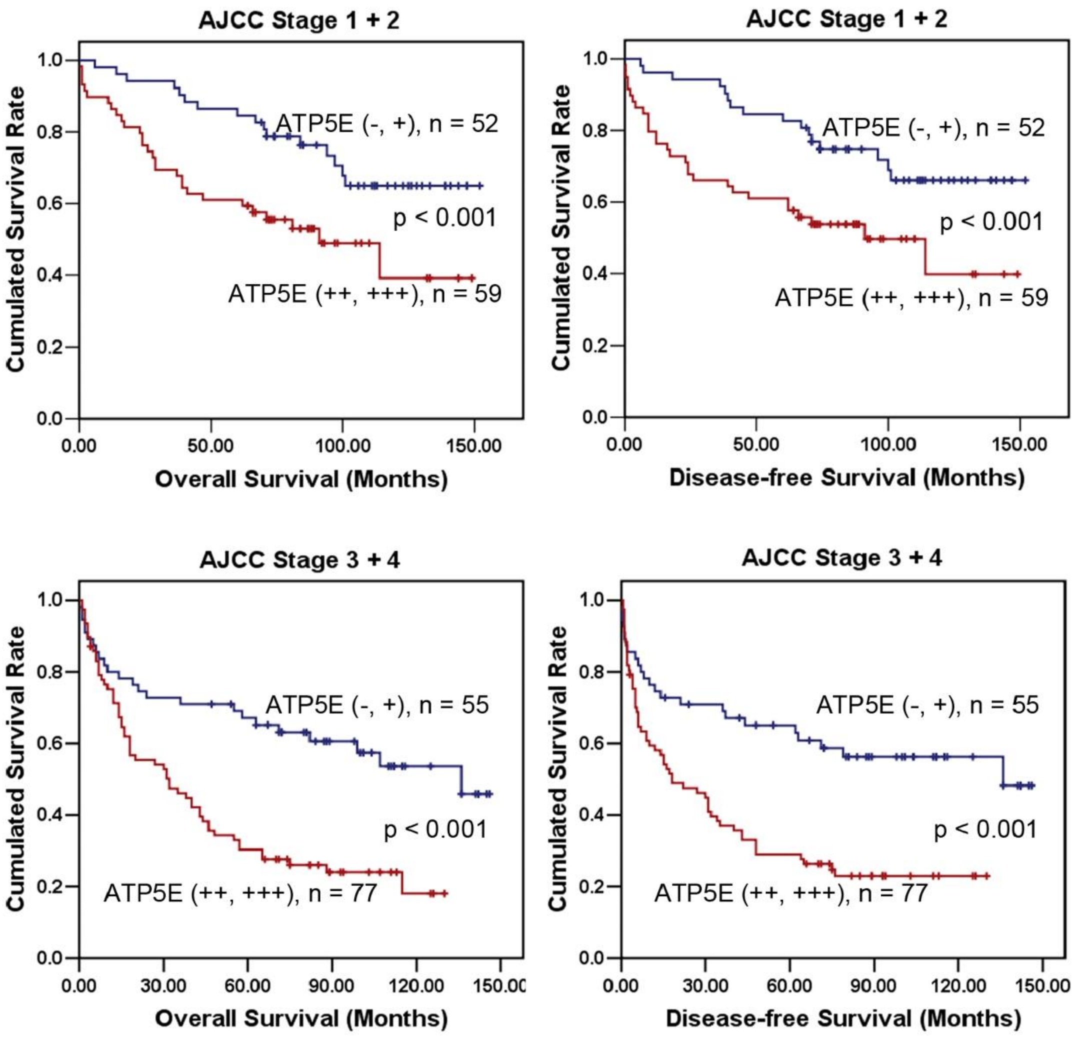

Figure S1. Kaplan-Meier analysis of overall and disease-free survival in stage I-II and stage III-IV colon cancer patients, stratified by ATP5E expression. ATP5E is significantly associated with disease-free survival and overall survival.

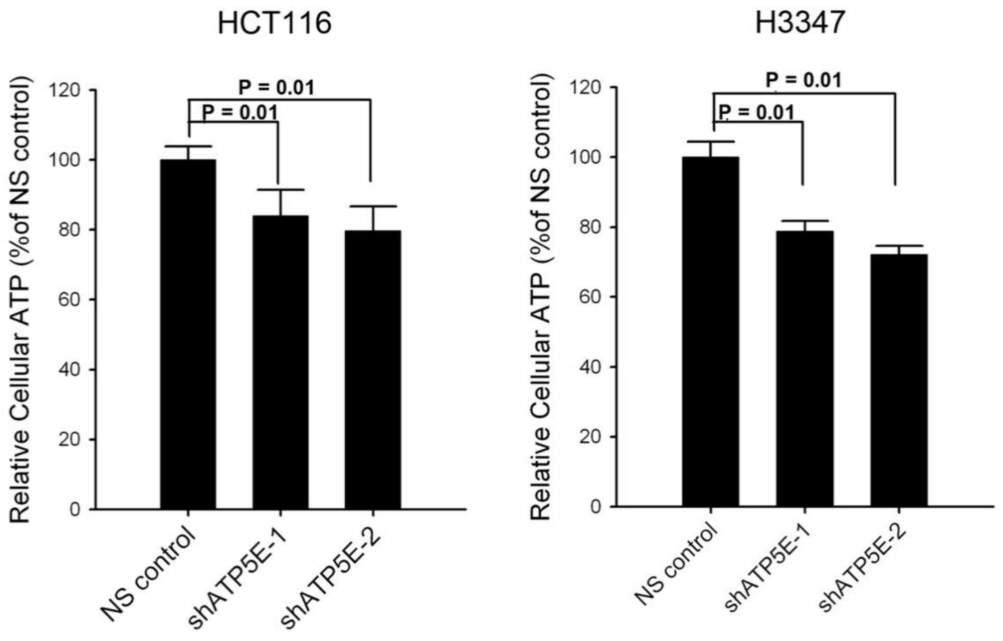

Figure S2. Relative intracellular ATP level upon ATP5E knockdown in HCT116 and H3347. Down-regulation of ATP5E, encoding a subunit of mitochondrial ATP synthase, significantly affects the formation of ATP (P=0.01).

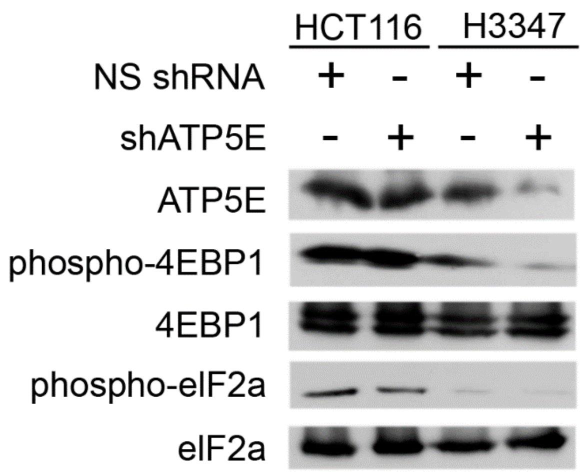

Figure S3. Western blot analysis of total/phosphorylation form of 4EBP1 and elF2a expression upon ATP5E knockdown in colorectal cancer cells, respectively.

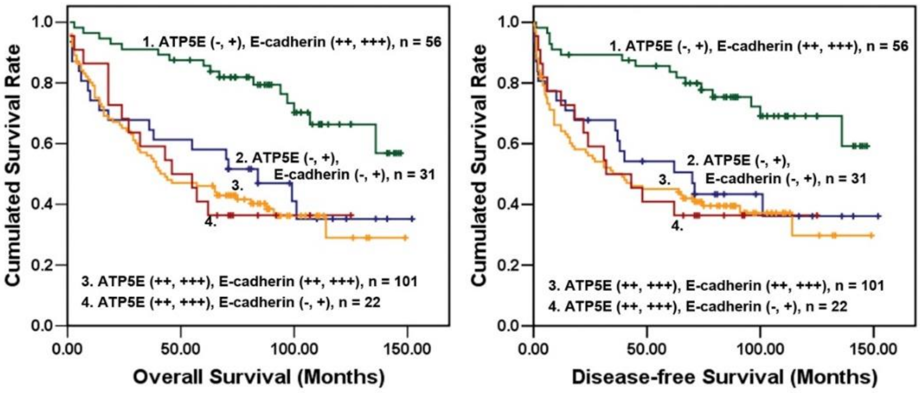

P-value	1	2	3	4
1	1	< 0.001	< 0.001	< 0.001
2		1	0.505	0.582
3			1	0.969
4				1

P-value	1	2	3	4
1	1	< 0.001	< 0.001	< 0.001
2		1	0.570	0.626
3			1	0.967
4				1

Figure S4. Kaplan-Meier analysis of overall and disease-free survival in combination with ATP5E status and E-cadherin status. ATP5E and E-cadherin expression are in reciprocal relationship. E-cadherin expression was frequently downregulated with concurrent overexpression of ATP5E in patients with poor disease-free survival and overall survival.

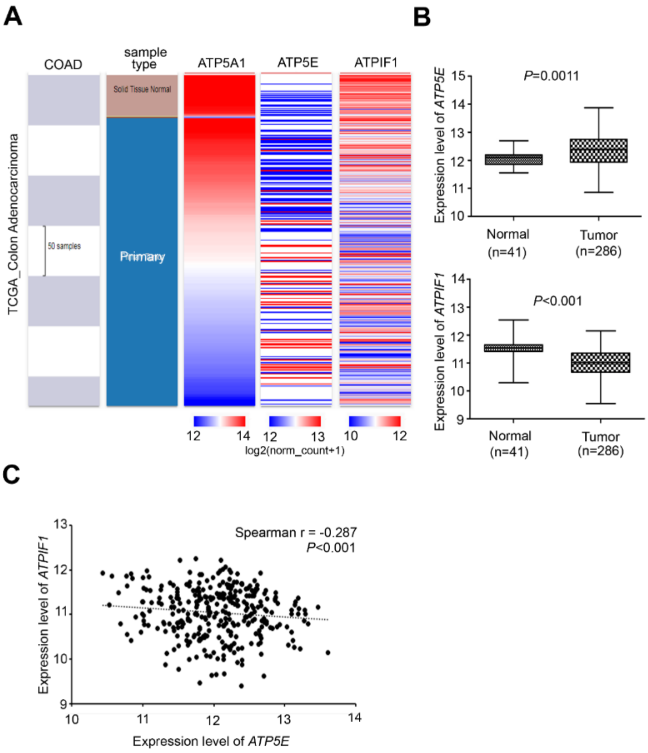

Figure S5. (A) Heatmap showed the expression of ATP5A1, ATP5E and ATPIF1 in the TCGA colon adenocarcinoma clinical cohort. (B) Quantitation the expression level of ATP5E and ATPIF1 in normal adjacent tissues and primary tumor group, respectively. (C) Correlation plot performed the significant negative association between ATP5E with ATPIF1.
